# Traffic noise inhibits inhibitory control in wild-caught songbirds

**DOI:** 10.1016/j.isci.2023.106650

**Published:** 2023-04-12

**Authors:** Christopher N. Templeton, Amber O’Connor, Sarah Strack, Franco Meraz, Katri Herranen

**Affiliations:** 1 Department of Biology, Pacific University, Forest Grove, OR 97116, USA

**Keywords:** Environmental science, Ethology, Animal science

## Abstract

Anthropogenic noise is ubiquitous across environments and can have negative effects on animals, ranging from physiology to community structure. Recent work with captive-bred zebra finches demonstrated that traffic noise also affects cognitive performance. We examined whether these results extend to animals that have experienced noise in the wild. We collected black-capped chickadees from areas frequently exposed to road traffic noise and tested them on a detour reaching task, a commonly used measure of inhibitory control. Those chickadees exposed to traffic noise playback had much lower performance on the task than control birds, indicating that noise negatively impacts inhibitory control. These data corroborate previous findings in lab-reared zebra finches. Furthermore, these results suggest that prior experience with traffic noise is not sufficient for animals to habituate to noise and overcome its negative effects on cognitive performance. Instead, noise-induced cognitive effects might have broad impacts on animal species living in noise-polluted habitats.

## Highlights

• Traffic noise pollution has a variety of negative impacts on animals
• Recent work suggests that noise reduces cognitive performance in captive-bred birds
• We show that cognition is also decreased in chickadees familiar with traffic noise
• Animals are unlikely to overcome noise-induced cognitive effects through habituation

## Graphical abstract

**Figure:**
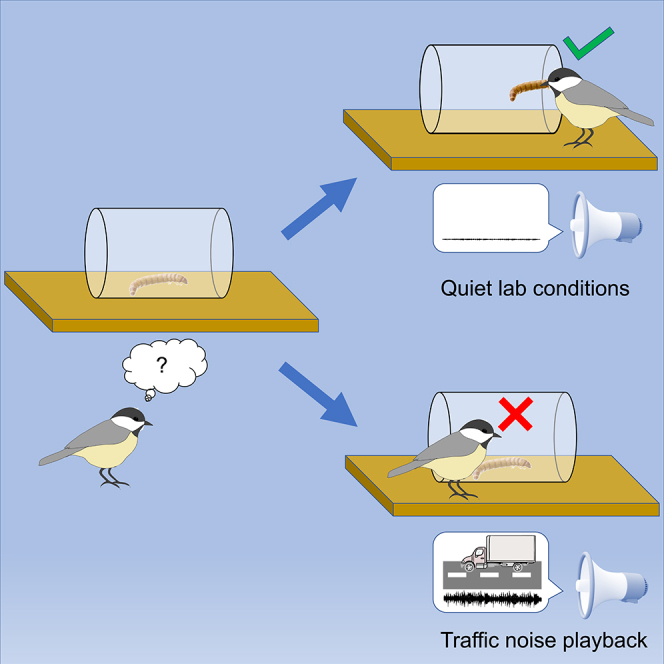

## Introduction

Rapid human population growth rates have driven a dramatic increase in anthropogenic noise pollution, which can have a variety of negative impacts on wildlife.^1^^,^^2^ Sound can directly impact animal anatomy and physiology, for example high-intensity sound waves can physically damage animal auditory systems.^3^ Sound can also indirectly impact animals, by influencing their behavior, and while these effects can be less obvious, they can still have significant impacts on individuals, populations, and communities. For example, because many animals use sound to communicate information, anthropogenic noise has been widely shown to negatively impact animal behavior by masking acoustic signals related to sexual reproduction,^4^ begging calls,^5^ and alarm calls.^6^^,^^7^ Noise pollution modifies animal stress response, gut microbiome, reproductive success, habitat use, and predator-prey interactions, suggesting that the acoustic environment can influence both individual fitness and population structures.^1^^,^^8^^,^^9^^,^^10^^,^^11^

In addition to the ecological effects of noise pollution, noise can also impact cognitive processing. These impacts were first observed in humans, with several studies suggesting that individuals in noisy environments, for example children attending school near airports, tended to perform poorer on cognitive assessments.^12^^,^^13^ While this topic has been less studied in animal systems, there is recent evidence that noise also impacts cognitive performance in animals. Prolonged playback of high-amplitude, white noise produced oxidative tissue damage in the brains of mice, in turn reducing their cognitive performance.^14^ Chronic noise exposure also causes stress and sleep disturbances, in mice and rats, respectively, both of which in turn impact cognitive processes.^15^^,^^16^ Experimental playback of noise during development resulted in decreased song learning accuracy in zebra finches (*Taeniopygia guttata*), with further impacts on immune function^17^ and smaller brain regions related to vocal learning.^18^ In addition to damaging brains’ anatomy and physiology, noise could impact cognitive performance by distracting animals from other tasks^19^^,^^20^ or increasing vigilance.^21^^,^^22^^,^^23^ Thus, anthropogenic noise pollution could have cognitive impacts in animals similar to those observed in humans.

Recent research indicates that anthropogenic noise pollution may have substantial negative impacts on avian cognitive processes. Osbrink et al.^24^ played road traffic noise at realistic amplitudes to captive-bred zebra finches and demonstrated that just hearing traffic noise significantly reduced cognitive performance on a variety of different foraging tasks. While zebra finches are an exceptionally useful study system that has been used to address many questions related to perception and learning,^25^ they are also domesticated lab animals that have been in captivity for over a hundred generations^26^ and it is therefore important to determine how generalizable previous results are to other species.^27^^,^^28^ Furthermore, although the Osbrink et al.^24^ findings suggest the potential for widespread impacts of noise on cognitive processes in animals, we know little about how these results actually translate to animals living in the real world, where noise exposure can vary across both space and time.

To begin examining the extent to which anthropogenic noise impacts cognitive processing in other animal species, we expand this line of research by focusing on another small songbird, the black-capped chickadee (*Poecile atricapilus*). Chickadees are an ideal study system for this type of research for a number of reasons. First, they are common and widely distributed throughout much of North America, where they frequently inhabit both urban areas and roadside habitats as well as more natural habitats.^29^ Next, cognitive behavior of chickadees, and closely related species, has been heavily researched, both in the laboratory and in natural environments.^30^^,^^31^^,^^32^^,^^33^ Last, it has been suggested that their cognitive ability and cognitive flexibility have allowed chickadees to succeed in novel environments, including urban and urbanizing areas.^34^ Previous work has shown that chickadee communication is negatively impacted by noise,^35^^,^^36^^,^^37^ but the impact of this noise on their cognition has not been studied.

Here, we use wild-caught chickadees to test whether the negative impacts of traffic noise on cognitive processing observed in zebra finches^24^ are generalizable to other songbirds. We presented chickadees with a detour reaching task that has been broadly used across human and animal systems to investigate inhibitory control, the ability to inhibit prepotent but ultimately counterproductive behavior.^38^ Inhibitory control is related to brain size, correlates positively with behavioral flexibility, and underlies other types of learning.^39^^,^^40^^,^^41^^,^^42^ This measure, therefore, has been suggested to provide a useful window into problem solving, planning, and flexibility,^39^^,^^41^ important traits for adapting to changing environments, such as those presented in human-dominated urban environments.^43^^,^^44^ We collected chickadees from locations near moderately busy highways to ensure that they had prior experience with traffic noise in the wild and tested them on a detour reaching task in the lab under experimentally manipulated levels of noise. If the patterns observed in Osbrink et al.^24^ are generalizable to other songbirds, we predicted that experimentally induced traffic noise exposure would also decrease cognitive performance observed in chickadees. Furthermore, the magnitude of difference between control birds and noise-exposed birds compared with previous work on captive-bred zebra finches should provide insights into the ability of animals to habituate to noise pollution with prior experience, potentially mediating its negative cognitive effects. If prior experience with traffic noise allows chickadees to overcome the negative effects of noise pollution, then we predict that the difference in cognitive performance between control and noise-exposed birds should be reduced compared with that observed in zebra finches. If habituation to prior noise pollution does not enable animals to overcome the negative cognitive effects of noise, then we expected to find patterns similar to those previously observed in captive-bred zebra finches.

## Results

**Figure 1:**
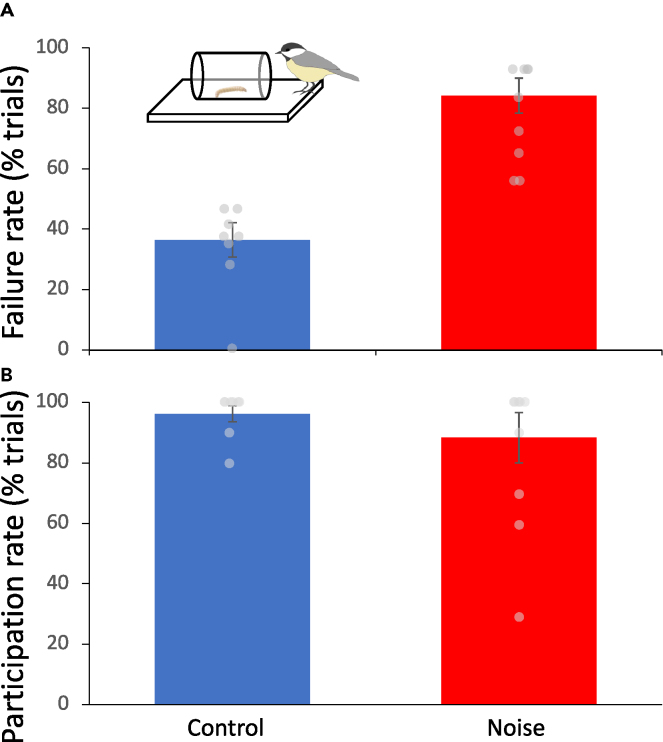
Traffic noise negatively impacts inhibitory control in black-capped chickadees (A) Birds hearing traffic noise playback were more than twice as likely to fail trials on a detour reaching task, although (B) their participation rate did not significantly differ from control birds. Data are represented as mean ± SEM, with individual data points depicted for each bird.

The presence of experimental traffic noise significantly decreased the performance of birds on the detour reaching task (t_15_ = 5.85, p < 0.0001, Cohen’s d = 2.83). Those birds performing the task in the presence of traffic noise were more than twice as likely to fail a given trial (mean ± SEM: 84.20 ± 5.8% failed trials compared with 36.5 ± 5.7% failed for control birds; Figure 1), by pecking at the clear cylinder before detouring to the open end. Control birds rapidly learned to inhibit their pecking response and detour around the clear cylinder, beginning to consistently solve the task after just a few trials (mean ± SEM: 4.5 ± 0.68 trials to the first of three successes in a row). In contrast, while birds exposed to experimental traffic noise did solve the task, none of the individuals managed to achieve the criteria of three successful trials in a row.

In contrast to trial performance, participation rates did not differ significantly between treatments (t_15_ = 1.47, p = 0.172, Cohen’s d = 0.682). Participation rates were generally high for all birds, with control birds participating in 96.25 ± 2.63% of trials compared and noise-exposed birds participating in 83.33 ± 8.33% of trials.

## Discussion

Chickadees exposed to experimental traffic noise had reduced performance on a detour reaching task. Compared with individuals tested under quiet control conditions, those birds exposed to noise were less than half as likely to correctly solve the task. In contrast, participation rates, a likely correlate of neophobia and stress level, did not significantly vary between treatments. These results indicate that the negative impacts of traffic noise on cognitive processing previously observed in zebra finches extend to detour reaching performance in wild-caught chickadees.

Given that performance on detour reaching has been shown to be a reliable indicator of the behavioral flexibility and problem-solving capabilities,^42^^,^^45^^,^^46^^,^^47^^,^^48^ our results suggest that anthropogenic noise could negatively affect black-capped chickadees inhabiting noise-polluted environments in the wild. Previous work has demonstrated that chickadees change their behavior in the presence of noise, altering vocalizations,^37^ vigilance and foraging,^49^ and use of alarm signals.^35^^,^^50^^,^^51^ Closely related mountain chickadees (*P. gambeli*) shift home ranges away from noise sources when exposed to traffic noise in the wild.^52^ Despite these effects on behavior, chickadees and other Parids have relatively highly developed cognitive abilities,^32^^,^^53^^,^^54^^,^^55^ which have helped them survive in a variety of human-dominated landscapes, such as urban areas and those near roads.^29^ These environments typically provide novel food sources, such as bird feeders, roadkill carrion, or garbage containers, and the ability of chickadees to effectively exploit these novel problems has helped them succeed in these environments. Yet, our results demonstrating diminished performance on detour reaching suggest that some of the very cognitive traits that help chickadees and other common urban species^44^^,^^56^^,^^57^ adapt to human-dominated environments could be negatively affected by anthropogenic noise. Does noise pollution counteract these species’ cognitive adaptations or have disproportionate effects on more cognitively advanced species? Better understanding how noise impacts detour reaching could provide a useful window into the cognitive flexibility necessary for chickadees and other species to overcome the challenges posed by urban areas and other human-dominated environments.^58^^,^^59^^,^^60^

The specific mechanisms driving noise-induced cognitive inhibition are not yet clear. Noise pollution is known to induce a stress response in animals^61^^,^^62^^,^^63^^,^^64^ and is associated with increased vigilance^2^^,^^21^ and it is possible that these could negatively impact cognitive performance despite likely being adaptive responses. Furthermore, a change in foraging behavior, such as avoiding foraging tactics that pose higher risk of predation, could be adaptive under noisy conditions. While we have not measured stress levels or vigilance behavior in this study directly, it seems likely that these would impact the degree to which animals participate in trials in addition to cognitive performance. Yet, we observed no significant effect of noise pollution on participation rates between experimental and control birds. This lack of difference in participation suggests that the impact of noise pollution on cognitive performance was not primarily driven by stress, increased vigilance, or other adaptive changes in behavior under noise. While the mechanisms in which noise pollution diminishes cognitive performance are not yet clear, another reasonable hypothesis would be that noise reduces attention to the task by acting as a distractor.^19^ Future work should further examine the mechanisms underlying noise-induced decreases in cognitive performance.

Our results suggest the potential for the cognitive effects of noise to translate to negative impacts on fitness of animals living in environments polluted with traffic noise. While there are many types of cognitive function potentially relevant to individual success,^65^ detour reaching could provide a useful window into the cognitive flexibility necessary to overcome new challenges in human-dominated environments. As detour reaching tasks assess the ability of an individual to inhibit their first instinct and instead find alternative solutions to a problem, the decrease in inhibitory control that our subjects demonstrated in the presence of traffic noise implies that traffic noise interferes with black-capped chickadees’ ability to quickly and efficiently solve problems. While the link between an individual’s cognitive ability and its fitness has been little tested, there is some evidence that cognitive ability predicts fitness. For example, Ashton et al.^66^ demonstrated that cognitive performance of female Australian magpies (*Cracticus tibicen dorsalis*) on a similar detour reaching and other cognitive tasks predicted reproductive success. Cole et al.^33^ showed that performance on novel problem-solving tasks also correlated with reproductive success in great tits (*Parus major*). Although the extent to which individual cognitive abilities impact fitness are still under debate,^65^ it seems likely that a significant reduction in cognitive performance due to noise pollution could affect an animal’s fitness, regardless of its initial cognitive abilities. In a natural environment polluted by traffic noise, the extra time and energy necessary to solve problems could be fatal, especially to small avian species like black-capped chickadees that have high metabolic rates.^67^ In addition to foraging-related cognition, it is likely that noise pollution could also impact other aspects of cognitive behavior in these and other animals. For example, song learning is generally thought to be a different cognitive module from the sorts of foraging tasks described here,^68^ but is also known to be impacted by anthropogenic noise pollution.^18^^,^^69^ Similarly, learning to discriminate among auditory stimuli, such as contact calls^70^ or sexual signals,^71^ are further examples of the potential for noise to disrupt a variety of cognitive processes in animals.

**Figure 2:**
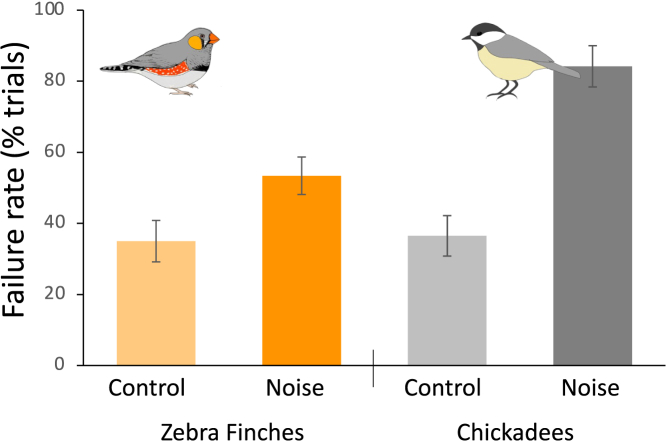
Wild-caught chickadee detour reaching performance compared with that of lab-reared zebra finches Despite control birds of each species having similar failure rates, chickadees exposed to traffic noise playback had higher failure rates than those observed in zebra finches. Data are represented as mean ± SEM percent of trials. Zebra finch data adapted from Osbrink et al.^24^

The findings with chickadees extend previous research demonstrating that lab-reared zebra finches have diminished cognitive performance when exposed to traffic noise.^24^ In addition to demonstrating that these noise-induced cognitive effects could be generalizable to other songbird species, comparing the magnitude of the effect could shed light on the ability of wild birds to adapt to noise pollution through habituation (Figure 2). Surprisingly, both chickadees and zebra finches participating in the detour reaching task under quiet control conditions had nearly identical performance levels, with failure rates of 36.5 ± 5.7% (chickadees) and 35.0 ± 5.8% (zebra finches). This suggests that on average, individuals of both species would exhibit similar levels of inhibitory control in the absence of noise pollution. However, chickadees exposed to noise pollution had much lower levels of performance (84.2 ± 5.8% failure rate) compared with zebra finches (53.4 ± 5.3% failure rate). Chickadee participation rates (control: 96 ± 3% and noise: 88 ± 8% of trials) were similar to those previously observed in zebra finches (control: 96 ± 4% and noise: 92 ± 4%; Osbrink et al.^24^), suggesting that noise had similar and relatively minor effects on neophobia for both species, despite differentially impacting cognitive performance between species. In addition to species differences, the birds used in these two studies had differences in their developmental history, with finches reared in the lab environment and chickadees reared in the wild and brought into the lab for testing. While chickadees were habituated to the lab environment before the experiment began and we observed no difference in performance of control birds compared with zebra finches, it could be that the testing environment was more novel for chickadees, heightening their vigilance levels so that the noise playback had a stronger impact. Another difference in rearing environment was the presence of noise pollution. Finches raised in the lab had little experience with traffic noise, though the chickadees were all collected from locations in which they would have previously heard traffic noise in the wild. The findings that noise playback had similar or even greater impact on chickadees that had previous experience with traffic noise suggest that any habituation to noise pollution developed through previous experience does not alleviate the effects of noise on animal cognition, at least in the way it was tested in this experiment. It will be important for future work to compare responses of chickadees from quiet environments to further understand the effects of potential habituation to noise pollution in this species. Quinn et al.^21^ demonstrated a similar lack of habituation to repeated noise playback in relationship to vigilance behavior, suggesting that animals might not be able to tune out noise pollution even with previous experience.

In addition to the prior experience and species of birds, other factors could potentially help explain the variation in effect size observed between the present data and the previous study.^24^ Chickadees used in this experiment were not provided with prior experience with detour reaching tasks following a protocol similar to Ashton et al.,^66^ whereas Osbrink et al.^24^ provided zebra finches with prior exposure to an opaque version of the task.^38^ Because chickadees are less neophobic, we elected to avoid the training phase, as it has been suggested that this prior exposure can make detour reaching results more challenging to interpret because success with the clear cylinder could result from inhibitory control or from a holdover of a previously learned rule from the prior training.^66^ Chickadees were given ten trials with the detour reaching task, compared with four trials (after training) in Osbrink et al.,^24^ which provided more opportunities for chickadees to learn about the cylinder and helped counteract for the lack of previous experience. Furthermore, if this difference in prior exposure was the major factor determining the outcome of the task, then we would have expected to observe species differences in the control group in addition to the noise group, which were not detected. It therefore seems likely that the differences in neophobia and prior training likely counterbalance each other between the two species, making the data reasonably comparable between experiments. Future work examining the degree to which different species respond to noise pollution would further our understanding of the implications of noise-induced cognitive decline for different species inhabiting anthropogenic landscapes.

### Limitations of the study

While this study has now shown that the effects of traffic noise exposure on cognitive performance extend beyond lab-reared birds, further research would be beneficial to better understand how noise pollution might affect cognitive behavior of wild animals. First, only one type of cognitive task—inhibitory control—was employed in this study. Inhibitory control is a good first measure because it is correlated with brain size,^38^ is related to planning, problem-solving, and judgment,^39^ and has been proposed to be a foundational ability that underlies many other types of cognition.^41^ However, the repeatability of this task is currently debated in the literature,^72^^,^^73^^,^^74^^,^^75^ so future work demonstrating repeatability of these scores for chickadees would be useful. Future research could also benefit from applying a battery of different tasks that assess different aspects of cognition, especially those cognitive components that are particularly relevant to the species of interest.^65^^,^^76^ For highly social animals with cognitive adaptations for caching, some of the most relevant tasks for chickadees might focus on assessing social learning and spatial memory.^54^^,^^77^^,^^78^ Future work examining the impact of traffic noise should ensure that the tasks are ecologically relevant to the biology of the particular study species being used.^65^^,^^76^ In addition to measuring performance on only one task, we only assessed birds with similar levels of prior exposure to traffic noise. All birds were collected from locations where they had previously been exposed to intermediate levels of traffic noise. While these results suggest that this level of previous exposure was not sufficient to habituate to the cognitive effects of traffic noise, it is possible that the effects we observed would vary for birds that had experienced different traffic noise regimes during development. For example, chickadees living in more highly urbanized environments are more tolerant of noise playback at feeding stations.^79^ It is also possible that animals habituate to very specific ambient noise profiles in their environments and if this is the case, the birds’ previous experience with noise would not necessarily generalize to help them overcome the experimental noise conditions used in this study. Future work examining birds originating from different noise environments, including habitats with both noisier and more acoustically pristine conditions, will better determine how noise regimes affect the cognitive impacts of noise pollution.

## STAR★Methods

### Key resources table

**Table:** 

REAGENT or RESOURCE	SOURCE	IDENTIFIER
**Experimental models: Organisms/strains**		

Black-capped chickadee (*Poecile atricapilus*)	Wild-caught in Washington County, OR USA	N/A

**Software and algorithms**		

SPSS, v. 28	IBM Corp, Armonk, NY	N/A

### Resource availability

#### Lead contact

Further information and requests for resources should be directed to and will be fulfilled by the lead contact, Chris Templeton (templeton@pacificu.edu).

#### Materials availability

This study did not generate new unique reagents.

### Experimental model and subject details

#### Study animals and housing

Wild black-capped chickadees were captured from locations on and within 3 km of the Pacific University campus in Forest Grove, Oregon USA (45.5204N, −123.1093W). All birds were collected in locations in which they would have been previously exposed to traffic noise from adjacent highways (80-300m from collection sites) and other roads that feature frequent (average daily vehicles traveling in each direction: 735 ± 354; ^80^) medium speed traffic from vehicles ranging from passenger cars to heavy logging trucks. While we did not collect specific data on the specific traffic noise profiles of these sites, they were generally similar to the playback stimuli (described below). A total of 17 individuals from four different winter flocks were captured using mistnets between February 2019 and March 2020. We were not able to accurately determine sex of the subjects because chickadees are sexually monomorphic,^29^ though like previous researchers,^54^ we had no reason to believe that males and females would perform differently on this task and did not deem it worth conducting the invasive tests necessary to determine sex. Exact ages were also unknown, but all birds were independent adults at the time of capture.

Birds were housed with their flock mates in a covered outdoor aviary on the Pacific University campus. The aviary was made from a wood frame with aviary netting and measured 2.3 x 3.8 x 2.2m high. The aviary contained numerous plants, perches, a roosting box, and fresh water provided from a pool with a fountain. Chickadees were given *ad lib* access to sunflower seeds, suet, and peanuts, with live mealworms also provided twice a day. Each bird was allowed to habituate to captivity for at least one week prior to testing and birds were kept in the aviary except when they were actively being tested.

#### Ethical statement

All work described in this paper was approved by Pacific University IACUC (910252) and conforms to ABS/ASAB guidelines for treatment of animals in behavioral research.

### Method details

#### Experimental preparation

To measure inhibitory control, we constructed a detour reach task that was based on the task used in numerous other studies with birds and other animals.^38^ The task consisted of a clear 11.5cm long x 6.3cm diameter tube made from thin plastic that was glued to a 13cm x 18cm wooden board.

We randomly split birds into two groups, those exposed to experimental traffic noise, and control birds receiving no noise playback. To ensure that we could compare our data directly with those data collected on zebra finches, we followed the detour reaching procedure established in Osbrink et al.^24^ and used the same noise playback stimuli found in that study. The chickadees in the traffic noise group received playback of road noise, which featured four exemplars of mostly continuous noise recorded from a moderately busy highway. To increase external validity, we used playback recordings that were derived from natural traffic recordings and presented at realistic sound levels.^17^^,^^24^^,^^81^ Recording details and complete noise profile information is included in Templeton et al.^6^, but briefly, recordings were made approximately 10 m from a rural two-lane highway in Germany with moderate levels of traffic and road noise using a Sennheiser ME66/K6 microphone and Marantz PMD660 digital recorder. We edited the recordings to create 30s playback files that included mostly constant noise using the ‘Mix Paste’ and ‘Crossfade’ functions in Adobe AUDITION 3.0 (Adobe Inc., San Jose, CA, USA). Traffic noise was broadcast from a Pignose 7-100 speaker (Pignose Amps, Las Vegas NV USA) located 1m from the focal bird’s cage that was calibrated with a Cel-246 SPL meter (L_AF_max setting, maximum sound pressure level, frequency weighting: A, time weighting: F; 20 *μ*PA reference value) so that the noise amplitude at the location of the bird was 70dB (to realistically simulate cars passing about 30m from the bird; ^6^ The speaker was also present for the control birds to control for any visual effect of the speaker, but no sound was played during these trials (ambient testing room amplitude ∼30dB).

#### Testing procedure

Individuals were captured from the aviary and placed in a testing cage (45 × 76 × 45 cm) located in an adjacent room that was acoustically and visually isolated from the other birds in the aviary. Each bird was given half an hour to habituate to the test cage with ad lib access to food and water. After habituation, food was removed for an additional 45 minute period to help increase motivation during the following experimental trials.

Prior to the trial, the bird was sequestered in one side of the cage using an opaque divider, so that the task apparatus could be placed on the other side of the cage out of view. Also out of view of the chickadee, mealworm halves were placed inside the clear tube of the detour reaching task at ¼, ½, and ¾ of the length of the tube, so that a chickadee needed to reach its head into either end of the tube to access a food reward and each bird could obtain up to 1.5 mealworms per successful trial. The tube was positioned in the side of the cage that did not contain the chickadee, oriented so that the side of the tube, not the openings, faced towards the chickadee when the divider was removed. We conducted 10 trials per chickadee, each lasting as long as it took for the subject to interact with the apparatus but not exceeding 5 minutes. Between each trial, there was a 5 minute break where the bird was again sequestered to one side and we restocked the mealworms in the task before the next trial. For the experimental subjects, traffic noise was played during the foraging trials, but not during breaks, following Osbrink et al.^24^

Trials were scored as successful if the bird detoured around to the open side of the tube without first attempting to obtain the mealworm through the side of the tube. If birds interacted with the side of the tube (e.g., pecking, flapping, scratching with feet, etc.) in an attempt to obtain the mealworm, we considered the trial a fail. If the subject made no attempt to obtain the mealworm during the 5 minute period, this trial did not count as a pass or fail and non-participation was excluded from the detour reaching score.

### Quantification and statistical analysis

#### Statistical analysis

To determine whether traffic noise impacted subjects’ inhibitory control, we compared the percent of trial successes and failures between the noise and control groups using an independent samples t-test. We tested a total of 8 birds in the control treatment and 9 birds in the noise treatment (n=17 birds). In addition to examining the percent of successful trials for each bird, we measured the number of trials required to become proficient with the task, as evidenced by successfully detouring on three trials in a row.^66^ In addition to comparing the detour reaching scores, we assessed participation rates of each group to ensure that any apparent differences in cognitive performance were not due to lack of participation. Again, we used a t-test to compare participation rates, but because the two groups did not have equal variances (Levene’s test, F=8.56, p=0.01), we used a Welch’s t-test which does not assume equal variances. All statistics were computed in SPSS v. 28.0 (IBM Corp, Armonk, NY) and all tests report two-tailed p values.

## Data Availability

• All data reported in this paper will be shared by the lead contact upon request. • This paper does not report original code. • Any additional information required to reanalyze the data reported in this paper is available form the lead contact upon request.
